# Sensitivity Analysis and Multi-Objective Optimization Strategy of the Curing Profile for Autoclave Processed Thick Composite Laminates

**DOI:** 10.3390/polym15112437

**Published:** 2023-05-24

**Authors:** Yiben Zhang, Guangshuo Feng, Bo Liu

**Affiliations:** School of Mechanical Engineering, University of Science and Technology Beijing, Beijing 100083, China; zyb1228@ustb.edu.cn (Y.Z.); fengguangshuo@ustb.edu.cn (G.F.)

**Keywords:** CFRP, curing process, sensitivity analysis, multi-objective optimization, finite element simulation

## Abstract

To mitigate the risk of manufacturing defects and improve the efficiency of the autoclave-processed thick composite component curing process, parameter sensitivity analysis and optimization of the curing profile were conducted using a finite element model, Sobol sensitivity analysis, and the multi-objective optimization method. The FE model based on the heat transfer and cure kinetics modules was developed by the user subroutine in ABAQUS and validated by experimental data. The effects of thickness, stacking sequence, and mold material on the maximum temperature (*T_max_*), temperature gradient (*ΔT*), and degree of curing (*DoC*) were discussed. Next, parameter sensitivity was tested to identify critical curing process parameters that have significant effects on *T_max_*, *DoC*, and curing time cycle (*t_cycle_*). A multi-objective optimization strategy was developed by combining the optimal Latin hypercube sampling, radial basis function (RBF), and non-dominated sorting genetic algorithm-II (NSGA-II) methods. The results showed that the established FE model could predict the temperature profile and *DoC* profile accurately. *T_max_* always occurred in the mid-point regardless of laminate thickness; the *T_max_* and *ΔT* increased non-linearly with the increasing laminate thickness; but the *DoC* was affected slightly by the laminate thickness. The stacking sequence has little influence on the *T_max_*, *ΔT*, and *DoC* of laminate. The mold material mainly affected the uniformity of the temperature field. The *ΔT* of aluminum mold was the highest, followed by copper mold and invar steel mold. *T_max_* and *t_cycle_* were mainly affected by the dwell temperature *T_2_*, and *DoC* was mainly affected by dwell time *dt_1_* and dwell temperature *T_1_*. The multi-objective optimized curing profile could reduce the *T_max_* and *t_cycle_* by 2.2% and 16.1%, respectively, and maintain the maximum *DoC* at 0.91. This work provides guidance on the practical design of cure profiles for thick composite parts.

## 1. Introduction

Thermosetting composites have been widely used in aerospace, automotive, civil engineering, and other fields due to their light weights, high specific strength, fatigue resistance, and strong designability [1,2,3,4]. The composites are usually produced by an autoclave with a determined cure cycle profile in order to initiate and sustain an irreversible cross-linking of the resin [5,6,7,8]. With the composite increasingly used in complex structures, the thickness would exceed the applicable range of curing parameters recommended by Manufacturer Recommended Cure Cycles (MRCC) [9,10,11]. It will inevitably lead to thermal gradients, overshoot, and insufficient curing or energy consumption [12,13]. Therefore, it is of great importance to analyze the curing process comprehensively and to develop an optimization approach.

Optimization strategies based on finite element numerical simulation are used as a general method to drive the appropriate cure profile for thick laminate. Most of the research is limited to single-objective optimization [14,15], and some used multi-objective optimization to strike a balance between process costs and product quality. For example, Yuan et al. [16] and Gao et al. [17] established a multi-objective approach to optimize the curing process for thick composites based on a multi-field coupled model with a surrogate model. The *t_cycle_* (cure time duration), *ΔT* (maximum temperature gradient), and *DoC* (degree of curing) using the optimal cure profile have been reduced by about 30.9%, 45.76%, and 16.88%, respectively, in comparison with the MRCC cure cycle. Tang et al. [18] introduced a multi-objective optimization method based on finite element simulation to control the *t_cycle_* and cure-induced defects of C-shaped composites. Compared with the original profile, the *t_cycle_* was shortened by 19% to 14,686 s. Similarly, Li et al. [19] proposed a method to optimize the fiber-reinforced composite injection molding process by combining the combined Taguchi response surface methodology and the NSGA-II approach. It indicated that NSGA-II was an effective method to solve the multi-objective optimization problem for the quality optimization of fiber-reinforced composite injection molding. Dolcum et al. [20] developed a novel approach based on the finite element method and a multi-objective genetic algorithm to optimize the cure profile for thick thermoset composites. The results showed that compared to the original curing profile, the optimized one led to approximately a 56% reduction of the maximum difference *DoC*, a 71% decrease in the maximum difference in *T_max_*, and a 33% reduction in *t_cycle_*. Struzziero et al. [21] also developed a method combining a finite element solution with a genetic algorithm to optimize the curing process of thick components. The optimized cured profile indicated improvements of about 70% in overshoot and a reduction in process time of about 14 h. However, the studies mentioned above rarely analyzed the material and structure effects on the thick composite curing process. Meanwhile, the problem that to what degree of curing profile parameters affected the simulation results also remains to be addressed.

The present work aims to comprehensively analyze the curing process of thick composites based on finite element simulation, parameter sensitivity analysis, and multi-objective optimization. Firstly, a FE model based on a heat transfer module and a cure kinetics module was developed for a thick laminate and validated by experimental data. The effects of composite thickness, stacking sequence, and mold material on *T_max_*, *ΔT*, and *DoC* were comprehensively discussed. Then, parameter sensitivity was tested to identify the critical process parameters which have significant effects on the curing results. Finally, a multi-objective approach to optimize the curing process for thick composites by integrating the RBF and NSGA-II algorithms was established. A decision-making method was also used to select the final optimal solution from the Pareto optimal set.

## 2. Finite Element Model

### 2.1. Thermo-Chemical Description of the Curing Process

The FE model included the heat transfer module and the cure kinetics module. The evaluation of the *DoC* and temperature is calculated by the thermo-chemical-coupled heat transfer module as in [22]:

(1) $$k_{\mathrm{xx}}\frac{\partial^{2}T}{\partial^{2}x}+k_{\mathrm{yy}}\frac{\partial^{2}T}{\partial^{2}y}+k_{\mathrm{zz}}\frac{\partial^{2}T}{\partial^{2}z}+\rho_{r}\left(1-V_{f}\right)H_{R}\frac{d\alpha }{\mathrm{dt}}=\rho_{c}C_{c}\frac{\partial T}{\partial t}$$

in which $\rho_{c}$, $C_{c}$ and $k_{\mathrm{ii}}\left(i=x,y,z\right)$ are the density, specific heat capacity, and thermal conductivity of the composite, respectively; $V_{f}$ is the fiber volume fraction in the composite. $\rho_{r}$ is the density of resin and $H_{R}$ is the total quantity of heat released from the curing reaction of a unit mass of the resin. T and t are the current temperature and curing reaction time, respectively. $\frac{d\alpha }{\mathrm{dt}}$ indicated the instantaneous *DoC*, which can be computed in the incremental step in FE analysis using the cure kinetics model of resin.

The curing kinetics model of resin is mainly divided into two kinds: the phenomenological model and the mechanism model. The phenomenological model used a single reaction to replace the whole reaction process; the mechanism model is more inclined to analyze the kinetic mechanism of the reaction process. Most researchers use phenomenological models to describe the curing reaction process of resin, and the expression of the curing kinetic model can be seen in [23,24]:

(2) $$\frac{d\alpha }{\mathrm{dt}}=\frac{{K\alpha }^{m}{\left(1-\alpha \right)}^{n}}{1+e^{C\left(\alpha -\alpha_{C}\right)}},{\;\alpha }_{C}=\alpha_{C0}+\alpha_{\mathrm{CT}}T,\;K={\mathrm{Ae}}^{\left(-\Delta E/\mathrm{RT}\right)}$$

in which K  is the activation energy; m and n are the first and second exponential constants, respectively; A is the pre-exponential coefficient; C and R are the diffusion constant and gas constant, respectively. $\alpha_{C}$ is the temperature-dependent DoC; $\alpha_{C0}$ is the constant at T = 0 K, and $\alpha_{\mathrm{CT}}$ is DoC increasing ratio with temperature.

The density of the composite $\rho_{c}$ and specific heat capacity of the composite $C_{c}$ can be calculated by the rule of mixture as in [25]:

(3) $$\rho_{C}=V_{f}\rho_{f}+\left(1-V_{f}\right)\rho_{r}$$

(4) $$C_{C}=V_{f}C_{f}+\left(1-V_{f}\right)C_{r}$$

in which $\rho_{f}$ and $C_{f}$ are the fiber density and specific heat capacity, respectively.

Similarly, the thermal conductivity of the composite $k_{ii}\left(i=x,y,z\right)$ can also be computed according to the rule of mixture as in [25,26]:

(5) $$k_{\mathrm{xx}}=V_{f}k_{f}^{xx}+\left(1-V_{f}\right)k_{r}$$

For unidirectional ply as the transversely isotropic material, in-plane thermal conductivity of composites perpendicular to the fiber direction ($k_{\mathrm{yy}}$) and in the thickness direction ($k_{\mathrm{zz}}$) are assumed to be equal and can be calculated as in [25,27]:

(6) $$k_{\mathrm{yy}}=k_{\mathrm{zz}}=k_{r}\left\{\left(1-2\sqrt{V_{f}/\pi }\right)+\frac{1}{B}\left[\pi -\frac{4}{\sqrt{1-B^{2}V_{f}/\pi }}\tan^{-1}\frac{\sqrt{1-B^{2}V_{f}/\pi }}{1+B\sqrt{V_{f}/\pi }}\right]\right\}$$

in which $B=2\left(\frac{k_{r}}{k_{f}^{\mathrm{yy}}}-1\right)$.

This research used the composite materials in Ref. [25] to conduct the finite element simulation. The thermal physical properties for 8552 resin and AS4 fiber as well as the cure kinetic constants for 8552 resin are listed in Table 1.

### 2.2. Finite Element Simulation

**Figure 1:**
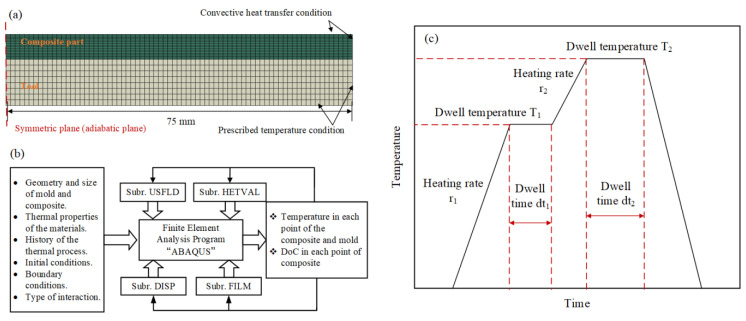
(**a**) Configuration for tool-composite assembly and corresponding boundary conditions in the FE model; (**b**) Flowchart of finite element simulation with subroutine; (**c**) Typical curing profile.

A cross-ply laminated composite plate with 5.4 mm thickness which consisted of 30 plies with stacking sequences of [90°_7_/0°_8_]_s_ was established in this study. It was cured on the invar mold as the experimental conditions. For model simplification and calculation efficiency, 1/4 part of the composite plate was modeled, which was 75 mm in width and length. The 8-node linear heat transfer brick (DC3D8) was for composite laminate and mold. The prescribed temperature boundary condition was set to be equal to the autoclave air temperature (Figure 1a).

In this paper, the commercial finite element software ABAQUS and FORTRAN subroutines are used to simulate the curing process of composite materials. The subroutines used include HETVAL, USDFLD, DISP, and FILM. HETVAL can define the reaction heat inside the composite; USDFLD is used to describe the curing degree field of the composite curing process; DISP is used to determine the temperature boundary conditions of the composite curing process, and FILM can determine the convective heat transfer boundary conditions of the composite curing process (Figure 1b).

The curing cycle was followed as in Ref. [25]. It can be divided into four stages: (a) the initial temperature was set to 25 °C; (b) the temperature was increased to 110 °C with a rate of 2.0 °C/min (*r_1_*); (c) the dwell temperature of 110 °C (*T_1_*) was held for 1 h (*dt_1_*); (d) the temperature was increased again to 180 °C with a rate of 2.0 °C/min (*r_2_*); (e) the dwell temperature of 180 °C (*T_2_*) was held for 2 h (*dt_2_*) and then (f) decreased to 25 °C at a rate of −2.0 °C/min (Figure 1c).

## 3. Parameter Sensitivity Analysis and Multi-Objective Optimization

### 3.1. Parameter Sensitivity Analysis

The typical curing profile of six parameters including the two heating rates (*r_1_*, *r_2_*), two dwell times (*dt_1_*, *dt_2_*), and two dwell temperatures (*T_1_*, *T_2_*) were extracted from the curing profile as the design variables.

Sobol sensitivity analysis is widely used in various fields in the industry to identify the most critical input variables and improve the accuracy and robustness of models. It was employed to quantify the impact of input variables on the output of a model or simulation and provided a measure of the relative importance of each input variable by decomposing the total variance in the model output into contributions [30,31]. Herein, the Sobol sensitivity analysis method was employed to investigate the effects of curing profile parameters on the results. The contribution of each parameter is derived based on variance decomposition. The system input-output function f(x) can be decomposed into a summary of increasing dimensions:

(7) $$f\left(x\right)=f_{0}+\sum_{i=1}^{n}f_{i}\left(x_{1}\right)+\sum_{i=1}^{n}\sum_{i\neq j}^{n}f_{i,j}\left(x_{i},x_{j}\right)+\cdots f_{123\cdots n}\left(x_{1},x_{2},\cdots ,x_{n}\right)$$

in which $f_{0}$ is the constant term, which is equal to the expectation value of the output. Each input variable $x_{i}$, i=1,2,⋯n is randomly distributed in the range of [0, 1].

The decomposed items in Equation (3) can be derived as the following functions:

(8) $$f_{0}=\int f\left(x\right)\mathrm{dx},\;f_{i}\left(x_{i}\right)=\int f\left(x\right){\mathrm{dx}}_{\sim i},\;f_{\mathrm{ij}}\left(x_{i},x_{j}\right)=\int f\left(x\right){\mathrm{dx}}_{\sim \left\{\mathrm{ij}\right\}}-f_{0}-f_{i}\left(x_{i}\right)-f_{j}\left(x_{j}\right)$$

in which $\int {\mathrm{dx}}_{\sim i}$ is the integration of all variables.

The total variance is defined as:

(9) $$D=\int_{0}^{1}f^{2}\left(x\right)\mathrm{dx}-f_{0}^{2}$$

The partial variances corresponding to the subset of parameters are defined as:

(10) $$D_{i_{1}\cdots i_{n}}=\int_{0}^{1}\int_{0}^{1}f_{i_{1}\cdots i_{n}}^{2}\left(x_{i_{1}},\cdots x_{i_{n}}\right){\mathrm{dx}}_{i_{1}}\cdots {\mathrm{dx}}_{i_{n}}$$

Under the case that the input variables are mutually orthogonal, the variance decomposition can be derived as

(11) $$D=\sum_{i=1}^{n}D_{i}+\sum_{1\leq i\leq j\leq n}D_{\mathrm{ij}}+\cdots D_{12\cdots n}$$

The Sobol sensitivity indices for a subset of parameters are defined as follows.

(12) Si1i2⋯in=Di1i2⋯inD 

### 3.2. Multi-Objective Optimization

The composite laminates are typical an-isotropic materials. The heat transfer coefficients are different along the fiber direction and in-plane as well as along the out-of-plane vertical fiber direction. A large amount of cross-linking reaction heat cannot be eliminated in time. The temperature overshoot phenomenon would occur and result in an uneven temperature field inside the laminate and incomplete curing of the components. To optimize the curing profile of thick composite laminates, the first and second dwell temperatures *T_1_* and *T_2_*, the first and second dwell times *dt_1_* and *dt_2_*, and the first and second heating rates *r_1_* and *r_2_* were used as designed variables. To minimize the *T_max_* and the curing profile time, *t_cycle_* was the optimization object. Meanwhile, to ensure that the overall curing of the composite material components was complete after the curing stage was completed, the minimum value of *DoC* should be greater than 0.9:

(13) $$\begin{array}{c} \mathrm{Find}\;X=\left({\mathrm{dt}}_{1},{\;\mathrm{dt}}_{2},{\;T}_{1},{\;T}_{2},{\;r}_{1},{\;r}_{2}\right)\\ {\mathrm{Min}\;T}_{\max},{\;t}_{\mathrm{cycle}}\\ S.T.\;\mathrm{DoC}\geq 0.9 \end{array}$$

**Figure 2:**
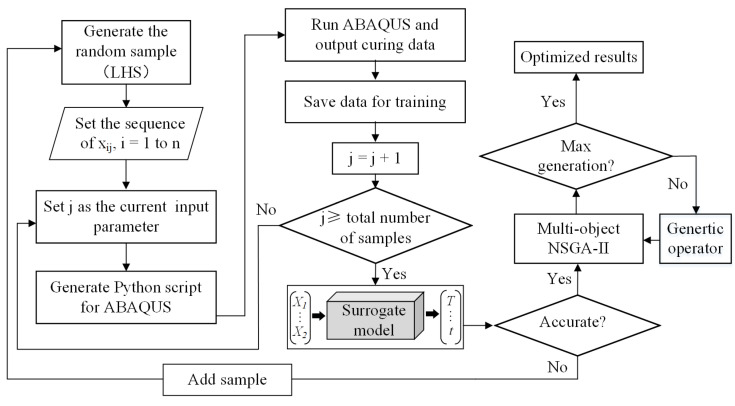
Flowchart of multi-objective optimization.

The steps of the multi-objective optimization design of the curing process were shown in Figure 2: Firstly, the Latin hypercube technique was used to generate random samples in the design space. Then the sample was re-organized to generate Python scripts for an efficient finite element simulation. The output values (T_max_, t_cycle_, DoC) from the FE model were saved for the surrogate model. Thirdly, the RBF was established as the surrogate model due to its applicability for higher-order nonlinear and multi-variable problems. Finally, NSGA-II multi-objective optimization was conducted. The radial basis function (RBF) maps the inputs to an output value based on its distance from a center point, which can be used to approximate complex functions and provide a good balance between model complexity and accuracy. Non-dominated Sorting Genetic Algorithm-II (NSGA-II), an extension of the original NSGA algorithm, is based on the concept of non-dominated sorting and is used to rank the solutions according to their dominance relationships.

## 4. Results and Discussion

### 4.1. Validation of the Finite Element Model

**Figure 3:**
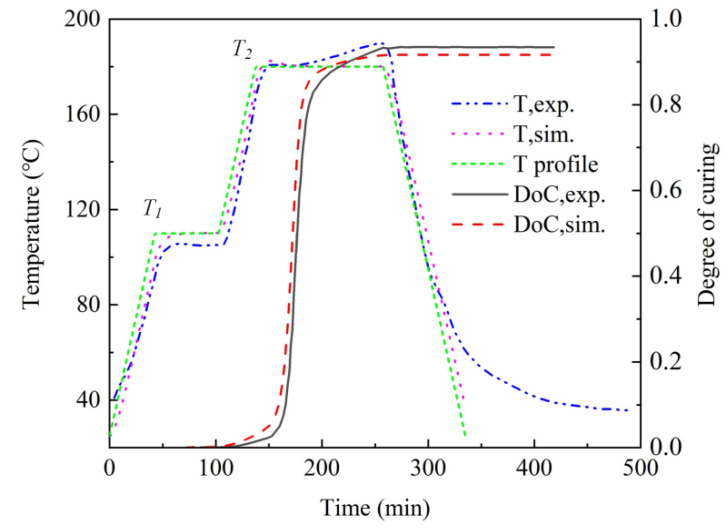
Comparison between experimental and numerical results.

The mid-point temperature profile and DoC profile from numerical simulation and experiments in Ref. [25] were compared in Figure 3, and the values were listed in Table 2. It can be seen that the simulation results agreed well with the experimental results. The values of dwell temperature T_1_ from the temperature profile, finite element simulation, and experiment were 110 °C, 110 °C, and 104 °C. The relative error between the experimental result and the numerical result was 5.8%. The values of dwell temperature *T*_2_ from the temperature profile, finite element simulation, and experiment were 180 °C, 181 °C, and 180 °C. The relative error between experimental and numerical results was 0.6%. The maximum *DoC* for experimental and numerical results was 0.93 and 0.91. The relative error was 2.2%. It can be concluded that the established finite element model was accurate and reliable for the following parameter sensitivity analysis and multi-objective optimization.

### 4.2. Parameter Sensitivity Analysis

**Figure 4:**
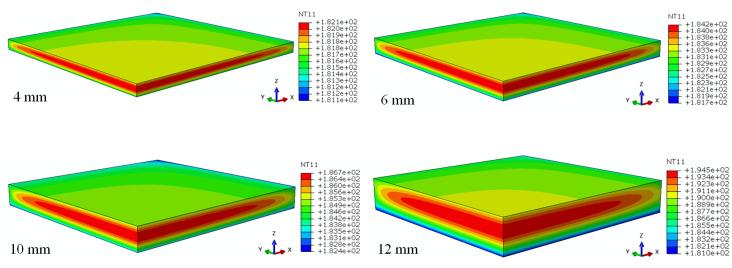
Cross-section temperature distribution of laminates with different thicknesses.

The 4 mm, 6 mm, 8 mm, 10 mm, and 12 mm thick laminates with [90_n_/0_n_]_s_ stacking sequence were used to evaluate the effect of thickness on the *T_max_*, *ΔT* (temperature gradient at the mid-point and surface-point), and *DoC* after the curing process. The cross-section temperature distributions of laminates with different thicknesses are shown in Figure 4. It can be seen that the maximum temperature always occurred in the mid-point regardless of the laminate thickness.

**Figure 5:**
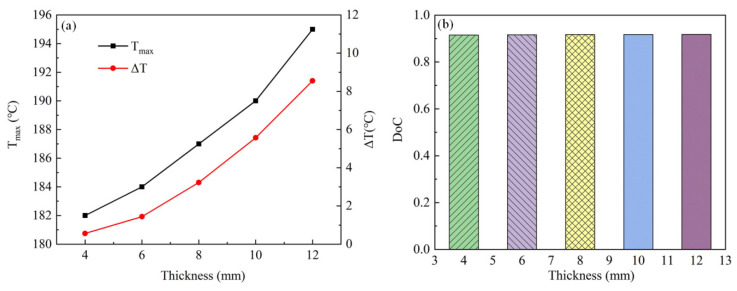
Effect of laminate thickness on (**a**) *T_max_* and *ΔT*; (**b**) *DoC*.

Figure 5 shows the effect of laminate thickness on curing results. The *T_max_* and *ΔT* increased non-linearly with the increasing thickness (Figure 5a), but the *DoC* was affected slightly by the laminate thickness (Figure 5b).

**Figure 6:**
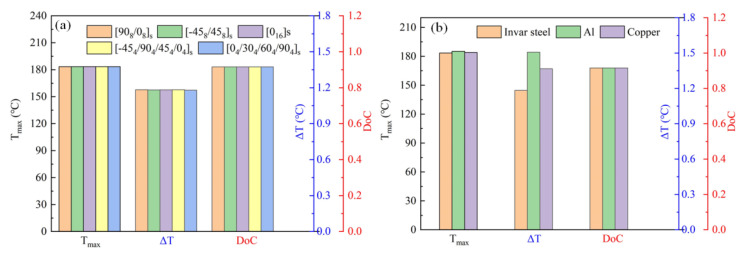
(**a**) Effect of stacking sequence on the curing process; (**b**) effect of mold material on the curing process.

The cross-ply, uni-direction, quasi-static, and angle ply is the most commonly used stacking sequence in the industry, and a helicoidal bio-inspired stacking sequence with excellent impact resistance has drawn more attention in recent days [32,33,34,35]. Thus, the laminates with [90_8_/0_8_]_s_, [45_8_/−45_8_]_s_, [0_16_]_s_, [−45_4_/90_4_/45_4_/0_4_]_s_, [0_4_/30_4_/60_4_/90_4_]_s_ were used to evaluate the effect of stacking sequence on the curing parameter. The results in Figure 6a showed that the T_max_, ΔT, and DoC were affected slightly by the laminate stacking sequence.

Invar steel, aluminum mold, and cooper mold were used to investigate the mold material on the curing result. The thermal parameter comparison of the three materials is listed in Table 3. The results are shown in Figure 6b. It was indicated that the mold material mainly affected the uniformity of the temperature field. The *ΔT* value of aluminum mold was the highest, followed by copper mold and invar steel mold.

### 4.3. Parameter Sensitivity Analysis

**Figure 7:**
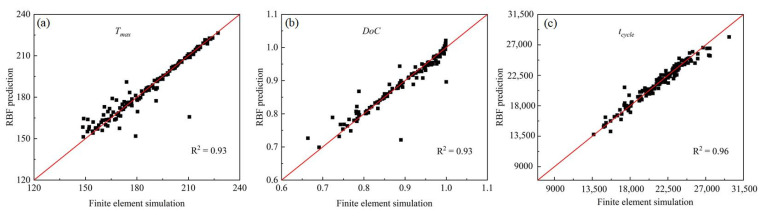
RBF prediction vs. finite element simulation on the (**a**) T_max_; (**b**) DoC; (**c**) t.

Parameter sensitivity analysis requires a large number of samples to support accurate variance analysis and provide reliable sensitivity information. Herein, RBF models were trained to serve as the surrogates of time-consuming FE simulation models. Figure 7 showed the scatter plot of finite element prediction and RBF surrogate model prediction. The red lines are the perfect fitting function. The more scatter points around the perfect fitting function, the higher the accuracy of the surrogate model. The coefficient of determination (*R^2^*) was used to evaluate the accuracy of the surrogate model. When *R^2^* equals 1, it indicated that all the scatters were located on the perfect-fitting line and that the surrogate model has the same prediction value as the FE model. As shown by the scatter plots in Figure 7, the surrogate models exhibit good consistency with the FE model in predicting *T_max_*, *DoC*, and *t_cycle_* since the scatters cluster around the perfect-fitting lines and the *R^2^* square value is above 0.9. These surrogate models will be used to support sensitivity analysis to identify the critical parameters that affect the simulation results of the curing process significantly.

**Figure 8:**
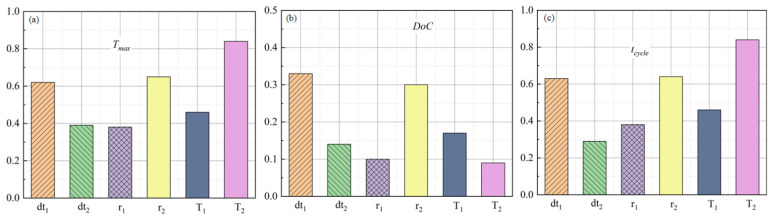
Curing sensitivity indices for (**a**) *T_max_*; (**b**) *DoC*; (**c**) *t_cycle_*.

Figure 8 provides the sensitivity analysis of *T_max_*, *DoC*, and *t_cycle_*. It was indicated that *T_max_* and *t_cycle_* were affected by the six parameters simultaneously as all the correlation factors were all higher than 0.29; meanwhile, the dwell temperature *T_2_* was the most significant process parameter, followed by dwell time *dt_1_* and temperature increasing ratio *r_2_*. *DoC* was mainly affected by dwell time *dt_1_*, temperature increasing ratio *r_2_*, and dwell temperature *T_1_*, and the influences of temperature increasing ratio *r_1_* and dwell temperature *T_2_* were not obvious as their correlation factors were less than 0.1.

### 4.4. Multi-Objective Optimization

**Figure 9:**
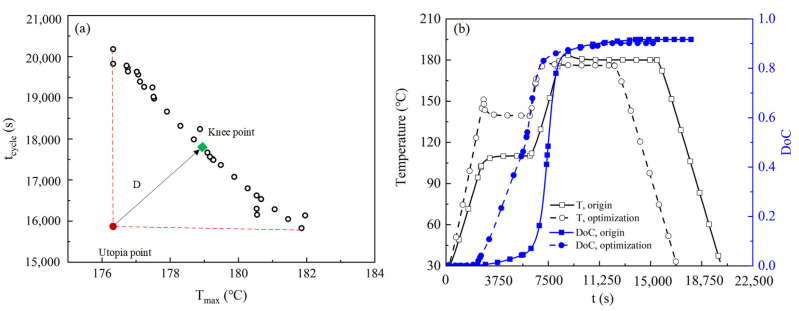
(**a**) Knee point closest to the ideal solution; (**b**) Development of the maximum and minimum temperatures and *DoC* using optimal and original curing profiles.

The optimized Pareto solution was shown in Figure 9. The two objectives, *T_max_* and *t_cycle_*, were opposite to each other, which means that a decrease in *t_cycle_* will inevitably lead to an increase in *T_max_*. Although Pareto frontiers can provide designers with many optimization solutions, designers can obtain desired solutions from different perspectives and obtain appropriate solutions. However, there are also designers who want to find an optimal and most satisfactory solution (Knee point) in the Pareto frontier, making each objective function as optimal as possible. In order to obtain the optimal solution from the Pareto front, the minimum distance method is used to select the Pareto front to obtain a Knee point. As shown in Figure 9a, the distance (Utopia point) of the ideal optimal solution from the Knee point is the smallest.

The *T_max_*, *t_cycle_*, and *DoC* from RBF were 178.9 °C, 0.91, and 17,667 s. Compared with the corresponding optimized values of 177.1 °C, 0.91, and 16,862 s from finite element simulation, the relative errors were 1%, 0, and 4.8%, which can be acceptable and prove the reliability of the surrogate model. The histories of temperature and *DoC* at the mid-point in the laminate derived by the optimized and original cure profiles are illustrated in Figure 9b. It can be seen that the maximum temperature at the laminate mid-point of the laminate by the original curing profile is 181 °C; the time cycle was 20,100 s; the *DoC* was 0.91. It indicated that the optimized curing profile could reduce the *T_max_* and *t_cycle_* by 2.2% and 16.1% and maintain the maximum *DoC.*

## 5. Conclusions

In this work, parameter sensitivity analysis and a multi-objective optimization were conducted using finite element simulation, the RBF surrogate model, and the NSGA-II algorithm. The finite element model based on the heat transfer module and the cure kinetics module was established and validated by experimental data. The effects of thickness, stacking sequence, and mold material on the *T_max_*, *ΔT*, and *DoC* were discussed. The curing profile parameter sensitivity was conducted to identify critical parameters that had significant effects on curing process, and a multi-objective optimization strategy was developed by combining optimal Latin hypercube sampling, the radial basis function (RBF,) and the non-dominated sorting genetic algorithm-II (NSGA-II) method. The effect of thickness, stacking sequence, and mold material on *T_max_*, *ΔT*, and *DoC*. The multi-objective optimization was conducted using *T_max_*, *DoC*, and *t_cycle_* as objectives. The final optimal solution for how to find from the Pareto optimal set has also been investigated. The main conclusions were as follows:

(1) The established FE model based on the heat transfer module and cure kinetics module can predict the temperature profile and *DoC* profile accurately. *T_max_* always occurred in the laminate mid-point regardless of the laminate thickness; the *T_max_* and *ΔT* increased non-linearly with increasing laminate thickness. However, the *DoC* was affected slightly by the thickness.
(2) *T_max_*, *ΔT*, and *DoC* were not changed obviously when compared to the finite element simulation results of laminate with cross-ply, uni-direction, angle-ply, and bio-inspired stacking sequence. The mold material mainly affected the uniformity of the temperature field. The *ΔT* of aluminum mold was the highest, followed by copper mold and invar steel mold.
(3) *T_max_* and *t_cycle_* were mainly affected by the dwell temperature *T_2_*; *DoC* was mainly affected by dwell time *dt_1_*, temperature increasing ratio *r_2_*, and dwell temperature *T_1_*. The multi-objective optimized curing profile showed good consistency with the surrogate model and could reduce the *T_max_* and *t_cycle_* by 2.2% and 16.1%, respectively, and maintain the maximum *DoC*.

## Figures and Tables

**Table 1:** Thermal physical properties and cure kinetic constants for composite materials [25,26,27,28,29].

Thermal Physical Properties for 8552 Resin and AS4 Fiber		Cure Kinetic Constants for 8552 Resin	
Parameter	Value	Parameter	Value
$k_{r}$ [W/(m.K)]	0.148 + 3.43 × 10^−4^·T	A (s^−1^)	7.0 × 10^4^
$k_{\mathrm{xx}}$ [W/(m.K)]	2.4 + 5.07 × 10^−3^·T	m	0.5
$k_{\mathrm{yy}}$ [W/(m.K)]	7.69 + 1.56 × 10^−2^·T	n	1.5
$C_{r}$ [J/(kg.K)]	931 + 3.47·T	R [J/(mol.K)]	8.314
$C_{f}$ [J/(kg.K)]	750 + 2.05·T	C	30
$\rho_{f}$ (kg/m^3^)	1790	$\alpha_{C0}$	−1.515
$\rho_{r}$ (kg/m^3^)	1300	$\alpha_{CT}$ (1/K)	5.171 × 10^−3^
$V_{f}$	57.4%	$H_{R}$ (J/kg)	5.74 × 10^5^

**Table 2:** Comparison between experimental and numerical results.

	T_1_	T_2_	DoC
Temperature profile	110 °C	180 °C	/
Numerical results	110 °C	181 °C	0.91
Experimental results	104 °C	180 °C	0.93

**Table 3:** Thermal parameters of mold material.

Material	Density (kg/m^3^)	Specific Heat (J/(kg °C))	Conductivity (W/(m °C))
Invar steel	8100	515	110.0
Aluminum	2800	880	174.6
Copper	8450	390	333.7

## Data Availability

Not applicable.
